# Experiences with Diagnosis and Treatment of Chagas Disease at a United States Teaching Hospital—Clinical Features of Patients with Positive Screening Serologic Testing

**DOI:** 10.3390/tropicalmed6020093

**Published:** 2021-05-31

**Authors:** Peter Hyson, Lilian Vargas Barahona, Laura C. Pedraza-Arévalo, Jonathan Schultz, Luisa Mestroni, Maria da Consolação Moreira, Matthew Taylor, Carlos Franco-Paredes, Esther Benamu, Poornima Ramanan, Anis Rassi, Kellie Hawkins, Andrés F. Henao-Martínez

**Affiliations:** 1 Department of Medicine, University of Colorado School of Medicine, Aurora, CO 80045, USA; peter.hyson@cuanschutz.edu; 2 Division of Infectious Diseases, University of Colorado School of Medicine, Aurora, CO 80045, USA; lilian.vargasbarahona@cuanschutz.edu (L.V.B.); jonathan.schultz@cuanschutz.edu (J.S.); carlos.franco-paredes@cuanschutz.edu (C.F.-P.); esther.benamu@cuanschutz.edu (E.B.); poornima.ramanan@cuanschutz.edu (P.R.); kellie.hawkins@dhha.org (K.H.); 3 Fundación Universitaria Juan N Corpas, 18301 Bogotá, Colombia; laurac-pedraza@juanncorpas.edu.co; 4 Adult Medical Genetics Program, Cardiovascular Institute, Division of Cardiology, University of Colorado School of Medicine, Aurora, CO 80045, USA; luisa.mestroni@cuanschutz.edu (L.M.); matthew.taylor@cuanschutz.edu (M.T.); 5 Faculdade de Medicina da Universidade Federal de Minas Gerais, Belo Horizonte-Minas Gerais 31270-901, Brazil; mariacvmoreira@gmail.com; 6 Hospital Infantil de Mexico, Federico Gomez, Mexico City 06720, Mexico; 7 Division of Cardiology, Anis Rassi Hospital, Goiânia 74110-020, Brazil; arassijr@cardiol.br; 8 Denver Health Medical Center, Denver, CO 80204, USA

**Keywords:** Chagas disease, trypanosomiasis, *Trypanosoma cruzi*, neglected tropical disease (NTD), pretransplant screening

## Abstract

Chagas disease (CD) is the third most common parasitic infection globally and can cause cardiac and gastrointestinal complications. Around 300,000 carriers of CD live in the U.S., with about 3000 of those in Colorado. We described our experience in diagnosing CD at a Colorado teaching hospital to revise screening eligibility criteria. From 2006 to 2020, we reviewed *Trypanosoma cruzi* (TC) IgG serology results for 1156 patients in our institution. We identified 23 patients (1.99%) who had a positive test. A total of 14/23 (60%) of positive serologies never had confirmatory testing, and 7 of them were lost to follow up. Confirmatory testing, performed in 9 patients, resulted in being positive in 3. One additional case of CD was identified by positive tissue pathology. All four confirmed cases were among patients born in Latin America. While most of the testing for CD at our institution is part of the pretransplant screening, no confirmed cases of CD derived from this strategy. Exposure risk in this population is not always documented, and initial positive results from screening are not always confirmed. The lack of standardized screening protocols for CD in our institution contributes to underdiagnosis locally and in health systems nationwide. Given a large number of individuals in the U.S. with chronic CD, improved screening is warranted.

## 1. Introduction

Chagas disease (CD), caused by the parasite *Trypanosoma cruzi*, is a public health problem of significance in Latin America, where it is endemic. It also affects other parts of the world via the immigration of affected individuals. The WHO estimates 6–7 million people are infected globally, most of whom reside in Latin America. WHO estimates CD causes approximately 10,000 deaths per year globally [1]. Current estimates, however, suggest a prevalence within the United States of up to 300,000 carriers of this disease [1,2]. CD has a global annual cost estimated at $627.46 million and 806,170 disability adjusted life-years, 10% of which is shouldered by the U.S. [3]. It has been called “the most important parasitic disease in the Western Hemisphere”, given the disease burden is 7.5× that of malaria [4].

The triatomine bug vector primarily transmits *Trypanosoma cruzi* by secreting the parasite in its urine and feces during blood meals. Other modes of transmission include food-borne, blood-borne (i.e., during blood transfusions), vertical maternal-fetal, and organ transplantation [1]. Acute infection is often asymptomatic. Most of the mortality and morbidity of this illness derives from long-term complications, especially cardiac disease, affecting 2% of asymptomatic infected patients annually [5]. CD epidemiology is changing, becoming a global issue due to improving control of vector-borne spread in endemic regions and increased migration of infected individuals [4]. While studies of blood donation screenings certainly imply a possible small role for vector-borne infection in the Southern United States, most affected individuals in the U.S. are born outside of the country [6]. One study screening Latin-American born individuals in Los Angeles found a prevalence of CD of 1.24% in this population, with Salvadorans having the highest prevalence [7]. Cardiac disease is also a growing problem in the United States. It increases hospital admissions, has significant requirements for advanced care, including pacemaker placement, mechanical support, transplant, and has an in-hospital mortality estimate of 3.2% [8]. Screening of Latin-American born patients with pacemakers in a U.S. clinic revealed a CD prevalence of 7.5%, with affected patients more likely to originate from El Salvador [9].

While the diagnosis of acute infection may be established with parasitologic methods, chronic CD is diagnosed with serology. A single serologic test is not sufficiently specific or sensitive for detecting an actual infection; thus, conventionally, diagnosis requires a positive result from two or more assays using different methods and detecting antibodies to different antigens [10].

Antiparasitic therapy decreases congenital transmission and an analytic decision model also showed that universal screening for CD among pregnant women in the U.S. would be cost effective [11,12]. Studies have shown that CD treatment reduced the risk of developing cardiomyopathy [13] and that asymptomatic screening of patients of Latin-American origin living in Europe is cost-effective [14,15]. However, no formal recommendations for screening for CD among asymptomatic high-risk patients in the United States exist (even those born in endemic areas). While diagnostic testing based upon clinical suspicion is insufficient to capture the significant disease burden of CD (given that many of those affected are asymptomatic), current screening protocols in transplant candidates also have low diagnostic yield. The predominant clinical and demographic characteristics of individuals diagnosed with a positive CD serology test in the Denver metropolitan area are unknown. Clinical preventive efforts have not evaluated the effectiveness of current diagnostic and treatment protocols for CD in this region.

In this case series, we review our experience of diagnosing and treating CD at a tertiary care hospital in Colorado. We aim to revise the eligibility criteria for screening and characterize the population with a positive Chagas serology.

## 2. Materials and Methods

The University of Colorado Hospital (UCH) Epic electronic health record (EHR) system was queried for diagnoses of CD and positive *Trypanosoma cruzi* IgG between 2006 and 2020. At our institution, a positive serological test for *T. cruzi*, usually enzyme-linked immunosorbent assay (ELISA) by ARUP lab (Salt Lake City, UT, USA), is followed up by a collection of a second blood sample, which is sent to the Centers for Disease Control and Prevention (CDC) for confirmatory testing with enzyme immunoassay (EIA) and *T. cruzi* excreted-secreted antigen blotting (TESA-blot) [16].

Cases were defined as patients with at least one documented positive serology for *Trypanosoma cruzi* IgG. Epic was also queried at the Denver Health Medical Center (DHMC), another metropolitan hospital that offers care to a large proportion of uninsured and underserved patients, but no additional cases were found. Demographic and clinical data were extracted by manual chart review. Data collected included age, sex, country of origin, comorbid medical conditions, initial indication for sending Chagas serology (i.e., diagnostic or screening), presence or absence of confirmatory diagnostic testing, type of disease (indeterminate, cardiac, digestive, or cardiodigestive), treatment, and follow up. Data were collected and analyzed using Microsoft Excel.

## 3. Results

**Figure 1:**
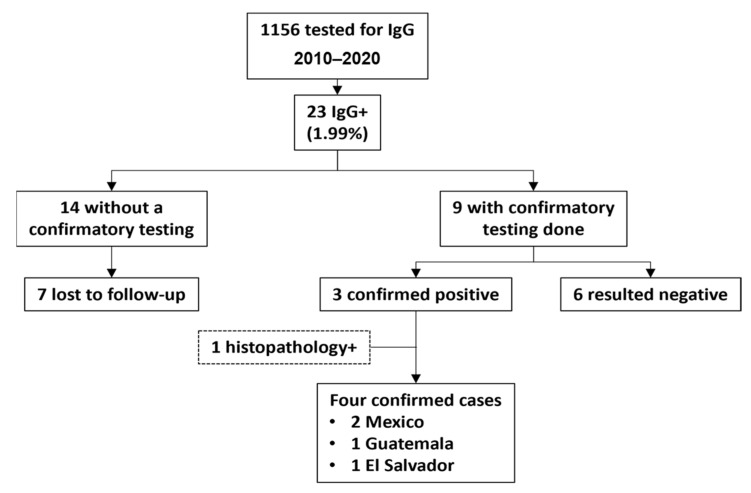
Detection and follow up of Chagas disease cases identified by serology at the University of Colorado Hospital from 2010 to 2020. Dotted frame: diagnosis established without serology testing.

The UCH Epic EHR showed that *Trypanosoma cruzi* IgG was ordered for 1156 patients, and only 23/1156 (1.99%) were positive from 2006 to 2020 (Figure 1). Demographic and clinical characteristics are provided in Table 1. Twenty-four cases were included in this series, 23 discovered through positive serology and one from positive molecular testing on biopsied tissue and serology (both conducted by the CDC). Eleven cases came from the solid organ transplant clinics, three from the bone marrow transplant clinic, two from the infectious disease clinic, two from the inpatient general medicine service, and five from the inpatient cardiology service. Most positive serologies (87%) were ELISA assays performed by ARUP laboratory. The remaining three serologies, all performed as part of screening panels prior to bone marrow transplant, were EIAs performed by Bonfils laboratory. Unless otherwise noted, confirmatory testing was performed by CDC as described in the methods section. 

The most represented comorbidities were congestive heart failure/cardiomyopathy and hypertension, with other common comorbidities including diabetes mellitus and chronic kidney disease. Thirteen subjects (54%) were born in CD endemic regions: 6 in Mexico, 3 in El Salvador, and 1 each in Honduras, Guatemala, Colombia, Bolivia.

Nine of twenty-three subjects with positive IgG had confirmatory serologic testing by a different assay, with 3/9 tests resulting positive. Overall, the diagnostic yield of *Trypanosoma cruzi* IgG serology was approximately 0.3%. One additional case of CD was identified by both positive tissue pathology and serology, bringing the total number of confirmed cases to 4, all of them among patients from Latin America.

Four cases were considered confirmed chronic CD. Three of these, as above, were diagnosed with positive *Trypanosoma cruzi* IgG, which was confirmed by repeat serology at the CDC. Two of the serologies were sent as part of the evaluation for cardiomyopathy, with Chagas cardiomyopathy diagnosed in both patients. The mode of transmission for these two patients was unclear. The third serology was a screening of a hospitalized woman from El Salvador with a history of renal transplant. She had received the kidney donation from her mother; thus, it is unclear if she had contracted the disease de novo or if the infection was donor-derived. She had indeterminate disease. The fourth confirmed case was a reactivation of CD in a heart transplant recipient. Excisional biopsy of an inguinal node had positive molecular testing for *T. cruzi.* Subsequently, tissue pathology from his native heart and *Trypanosoma cruzi* IgG serology were both positive at the CDC. His case would be considered a determinate cardiac disease with an unknown mode of infection. Neither of these post-transplant patients appears to have been screened for CD prior to transplant. All four CD cases were born in endemic regions (2 in Mexico, 1 in Guatemala, and 1 in El Salvador). Three (75%) of these cases were discovered as a result of diagnostic testing.

Six cases can be considered confirmed, “false positives” based on an initial positive IgG but negative confirmatory testing. Five of these cases received confirmatory serologic testing by the C.D.C. The sixth patient had an initial positive blood donation screen for *Trypanosoma cruzi* IgG serology, a positive EIA but a negative enzyme strip assay. Testing was followed by a positive ELISA and a negative immunofluorescence assay (I.F.A.). The ELISA was performed by ARUP and the IFA by Quest Diagnostics; further information about the other two assays (performed by the blood donation agency) could not be found [17]. In light of discordant tests, this was considered a false-positive result. Four of these false positives were sent as part of pretransplant screening and one as a diagnostic test in a patient with esophageal pathology and CD risk factors. Three of these false-positive patients (50%) were born in endemic countries.

**Figure 2:**
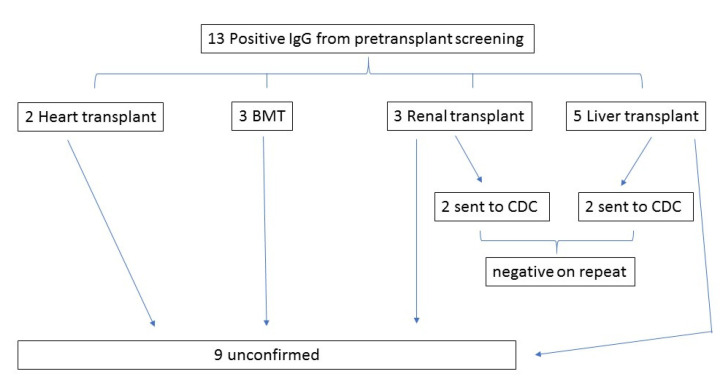
Follow up of pretransplant screening serologies.

Thirteen out of twenty-three positive serologies (57%) in this series were sent as part of the pretransplant screening, with only 4 of 13 (31%) followed with confirmatory testing (Figure 2). Organ transplant evaluations included three renal, five liver, two heart, and three pre-stem cell transplants for hematologic malignancy. Nine of thirteen (69%) positive serologies from the pretransplant screening were in patients from non-CD endemic countries. Indication for screening in patients not born in CD endemic countries (i.e., risk based on travel history) was only documented in two of nine cases. Notably, five patients who received pretransplant screening did not have a transplant infectious disease consult, and screening appears to have been sent automatically without a detailed travel history. No patients were diagnosed with chronic CD from pretransplant screening.

Overall a total of 14/23 (61%) positive screening serologies were never followed with confirmatory testing. Seven of these patients were lost to follow up. In one patient, the positive test result was not followed by further investigation. One patient declined further testing. Two patients seem to have had their positive results overlooked in the context of other terminal diseases, with one of these having confirmatory testing documented as “pending” but never ordered. Three patients had EIA sent for *Trypanosoma cruzi* IgG as part of a large serology panel prior to stem cell transplant. EIAs were sent multiple times in these subjects and were inconsistent (sometimes positive and sometimes negative), but no provider seems to have commented on these results in the chart, attempted to repeat serology with a different assay, or sent a sample to the CDC.

Three subjects with positive *Trypanosoma cruzi* IgG also had positive *Strongyloides* serology. Four were diagnosed with latent tuberculosis via the Quantiferon Gold assay.

## 4. Discussion

Our results indicated shortcomings in CD screening uniformity at our institution. Our results suggest that most screening for CD occurs as part of the pretransplant evaluation. Most patients with positive pretransplant *Trypanosoma cruzi* IgG were, however, (a) not from endemic countries and (b) never had confirmatory testing sent. There were no patients diagnosed with CD or treated based on pretransplant testing. Meanwhile, two patients (with risk factors) ultimately received a diagnosis of CD years after receiving solid organ transplantation.

We found a high proportion of false-positive *Trypanosoma cruzi* IgG (6/9) through confirmatory testing. Three of these patients had migrated from Chagas endemic countries (two from Mexico, one from Colombia). This high false positivity rate may reveal issues with the serologic test performance in a relatively low prevalence population. A prior study has shown geographic variability in serologic test performance with levels of antibody reactivity and clinical sensitivity lower in test subjects from Mexico than Central or South America, clearly a problem given the high proportion of CD carriers in the U.S. who come from Mexico [18]. This may fail to detect the infection from a single serology or result in discordance between two serologies.

Additionally, the necessity of collecting a fresh blood sample for repeat serology to confirm an initial positive test represents more opportunity for a patient to be lost to follow up. New generation testing is being developed, which uses large mixtures of recombinant antigens and multiple detection systems to achieve optimal sensitivity and specificity from a single test [19]. We look forward to the more widespread availability of such testing as current protocols represent a clear barrier to adequate diagnosis and treatment.

The majority of true positive results in this series resulted from diagnostic testing in the context of cardiac disease in individuals who had an epidemiologic risk factor. These patients were then treated with benznidazole. Unfortunately, the available evidence suggests that treatment efficacy in this context is guarded [20]. It would be far better to diagnose chronic CD at an earlier stage before developing cardiac manifestations, as treatment at that time would confer much more significant benefit. We suspect that a more standardized screening program for CD among at-risk populations would result in a higher diagnostic yield and more opportunities for treatment with long-term benefit. The available evidence indicates that screening for CD among asymptomatic patients could be reasonably undertaken during prenatal obstetric visits, and in light of higher pretest probability, could also be targeted to patients born in endemic countries who are seen at primary care clinics, cardiology clinics, or other settings [7,9,12]. Given a CD prevalence of around 0.05% in Colorado, the number needed to screen to find one case is about 285. The diagnostic yield would be expected to be higher if screening was targeted to individuals with epidemiological risk factors.

The American Society of Transplantation recommends Chagas serologic testing for transplant recipient candidates with epidemiologic risk [21]. Despite the apparently low diagnostic yield from screening in our case series, it is worth pointing out that CD screening is relatively inexpensive compared to organ transplantation, and the consequences of missing this diagnosis can be devastating. Often, many patients who undergo a pretransplant evaluation do not get listed for transplant and are no longer under the care of an infectious diseases provider. Many pretransplant patients live out of state and return to their local care providers for follow-up, making it challenging to send confirmatory testing to CDC. We consider that the selection of screening candidates based on exposure risk and follow up of positive results are integral to an effective screening program.

Neglected tropical diseases (NTDs) are defined as infections “strongly associated with poverty in tropical and subtropical environments [22]”. They disproportionately affect the most economically disadvantaged persons globally—the bottom billion [23]. Therefore, devoting attention to these NTDs represents an opportunity for remedying socioeconomic health inequity, both in the U.S. and abroad.

The United States has the most extensive case burden of CD of any nonendemic country. Nevertheless, this disease remains underdiagnosed [24]. Our review indicates that CD is similarly underdiagnosed in our healthcare system. Colorado is home to an estimated 271,404 persons born in Latin America, and its disease burden is thought to be over 3000 cases [2,25]. UCH, in Aurora, CO, USA is the largest quaternary care center in the Eastern Rocky Mountain region, serving as a local referral center for cases of CD in the greater Denver metropolitan area and a regional referral center for advanced heart failure, including Chagas cardiomyopathy. The Denver metro area, with more than 2.9 million residents (about the population of Connecticut), represents about half of the total population of the state. Thus over 1000 Chagas infected persons might be predicted to live in this area. Despite a significant expected CD case burden in the region served by UCH, our review only revealed four confirmed cases between 2006 and 2020. Reasons for underdiagnosis are likely numerous and include a lack of awareness of CD among front-line providers and resultant inadequacy of screening in the at-risk population, the asymptomatic nature of the disease, the inequity of the U.S. healthcare system and the fact that the at-risk population (primarily Latin American-born immigrants) is disproportionately lower-income or undocumented and has less access to health care [24,26].

Given the not insignificant incidence of coinfection with tuberculosis and strongyloidiasis observed in this study, we would recommend screening for these diseases in conjunction with Chagas testing. The United States Preventive Services Task Force (USPSTF) notes that foreign-born individuals constitute 66.2% of all latent tuberculosis infection (LTBI) diagnosed in the U.S., with Mexico among the five most represented countries of origin. Screening of individuals born in or former residents of countries with increased tuberculosis prevalence is recommended [27], and the USPSTF could develop similar screening recommendations for CD. The current COVID-19 crisis represents an opportunity to screen groups for NTDs, including Chagas, as populations at-risk are being disproportionately affected by COVID-19. Many such individuals are being brought in contact with the healthcare system for the first time [28].

This study has some limitations inherent to retrospective reviews. Cases were discovered and reviewed through search queries in the “slicer-dicer” platform in the Epic electronic health record but the search terms accepted were somewhat limited. While “Chagas IgG by EIA” could be searched, for instance, one could not search serologies by IFA or lateral flow assay (LFA). Many of the included subjects never received confirmatory testing, limiting the conclusions, which can be drawn about carriers of chronic CD (while also providing opportunities for systems-based measures to improve institutional screening protocols and confirmatory testing). Additionally, many records were incomplete since many of the subjects included in this descriptive case series were referred from other healthcare systems for care and subsequently lost to follow up. Several records were scanned in from the era preceding electronic health records and were thus incomplete. The actual sample size of positive CD serologies and confirmed cases were quite small, which limits the conclusions, which can be made about this population.

## 5. Conclusions

Despite a significant prevalence and rising cost to society, CD remains underdiagnosed in our healthcare system and the United States as a whole. Systematic attempts at screening patients based upon epidemiological risks might yield more actionable diagnoses, allowing for early treatment and prevention of transmission of CD. Special focus should be given to screening high-risk groups with potential exposure, including pregnant women, candidates for organ transplantation and potential organ and blood donors. Screening programs also need to include measures to ensure that confirmatory testing follows initial positive screens. Future efforts should be aimed at establishing and studying screening programs with established guidelines for inclusion as a proof of concept for the broader American healthcare system.

## Figures and Tables

**Table 1:** Characteristics of patients with positive *Trypanosoma cruzi* IgG ^1^.

	Confirmed Positive *n* = 3	Confirmed Negative *n* = 6	Confimatory Testing Not Done *n* = 14	Total *n* = 23
Median Age	55	64	57	59
Female	2 (66.7%)	4 (67%)	3 (21%)	9 (39%)
Born in CD endemic region	3 (100%)	3 (50%)	6 (43%)	12 (52%)
Hypertension	1 (33%)	4 (67%)	5 (36%)	10 (43%)
Cirrhosis	0	2 (33%)	3 (21%)	5 (22%)
CKD	1 (33%)	2 (33%)	4 (29%)	7 (30%)
Diabetes	2 (66.7%)	2 (33%)	3 (21%)	7 (30%)
Cardiomyopathy/CHF	2 (66.7%)	0	8 (57%)	10 (43%)
Arrhythmia	2 (66.7%)	0	5 (36%)	7 (30%)
GI Disease	0	1 (17%)	0	1 (4%)
Treated (Benznidazole)	3 (75%)	0	0	3 (13%)
Test Indication Screening	1 (33.3%)	5 (83%)	9 (64%)	15 (65%)
Test Indication Diagnostic	2 (66.7%)	1 (17%)	5 (36%)	8 (35%)

^1^ Confirmed: if second serology (by different assay/different antibody) performed in house or sent to CDC. Unconfirmed: if serology was not repeated. Not included: a case diagnosed with tissue pathology.

## Data Availability

The corresponding author had full access to data in the study and had final responsibility for the decision to submit the manuscript for publication. The datasets generated and analyzed in the current study are available from the corresponding author on reasonable request.
